# Allometry Between Vegetative and Reproductive Traits in Orchids

**DOI:** 10.3389/fpls.2021.728843

**Published:** 2021-10-13

**Authors:** Jing-Qiu Feng, Feng-Ping Zhang, Jia-Lin Huang, Hong Hu, Shi-Bao Zhang

**Affiliations:** ^1^Key Laboratory of Economic Plants and Biotechnology, Yunnan Key Laboratory for Wild Plant Resources, Kunming Institute of Botany, Chinese Academy of Sciences, Kunming, China; ^2^University of Chinese Academy of Sciences, Beijing, China; ^3^Yunnan Key Laboratory of Dai and Yi Medicines, College of Traditional Chinese Medicine, Yunnan University of Chinese Medicine, Kunming, China; ^4^Yuxi Normal University, Yuxi, China

**Keywords:** allometry, flower traits, inflorescence length, orchids, peduncle diameter, leaf traits

## Abstract

In flowering plants, inflorescence characteristics influence both seed set and pollen contribution, while inflorescence and peduncle size can be correlated with biomass allocation to reproductive organs. Peduncles also play a role in water and nutrient supply of flowers, and mechanical support. However, it is currently unclear whether inflorescence size is correlated with peduncle size. Here, we tested whether orchids with large diameter peduncles bear more and larger flowers than those with smaller peduncles by analyzing 10 traits of inflorescence, flower, and leaf in 26 species. Peduncle diameters were positively correlated with inflorescence length and total floral area, indicating that species with larger peduncles tended to have larger inflorescences and larger flowers. We also found strongly positive correlation between inflorescence length and leaf area, and between total floral area and total leaf area, which suggested that reproductive organs may be allometrically coordinated with vegetative organs. However, neither flower number nor floral dry mass per unit area were correlated with leaf number or leaf dry mass per unit area, implying that the function between leaf and flower was uncoupled. Our findings provided a new insight for understanding the evolution of orchids, and for horticulturalists interested in improving floral and inflorescence traits in orchids.

## Introduction

Floral display, which includes floral number, size, color, and arrangement, has a central influence on plant reproductive success (Harder and Johnson, 2005; Iwata et al., 2012). Plants with larger inflorescences and flowers receive generally more pollinator visits and increased reproductive success (Pleasants and Zimmerman, 1990; Harder and Johnson, 2005). Not surprisingly, the functional and evolutionary significance of flower and inflorescence sizes are subjects of strong interest (Darwin, 1859; Grant, 1950; Wyatt, 1982; Armbruster, 1996; Galen et al., 1999; Elle, 2004; Fenster et al., 2004; Strauss and Whittall, 2006).

Most studies have focused on the size-relationship between plant vegetative organs (Preston and Ackerly, 2003; Westoby and Wright, 2003; Sun et al., 2006; Yang et al., 2010; Fan et al., 2017). For example, various studies have shown that leaf and stem allometry are positively correlated (Preston and Ackerly, 2003; Westoby and Wright, 2003; Sun et al., 2006; Normand et al., 2008; Yang et al., 2010; Fan et al., 2017). These correlations imply that larger diameter branches can support large leaves mechanically and hydraulically (Niinemets et al., 2006; Normand et al., 2008).

Allometry is a useful integrative tool in zoology, indicating relationships between diverse measures, which has been widely used in plants (Western, 1979; Midgley and Bond, 1989). A previous study has shown that inflorescence size is allometrically related with leaf and stem size in *Leucadendron* and *Protea* (Proteaceae; Midgley and Bond, 1989). If these correlations are prevalent among flowering plants (Niklas and Enquist, 2003), reproductive organs may show similar correlations as those observed in vegetative organs. In other words, large-diameter stems can support larger inflorescences. However, such allometric correlation has been rarely tested on reproductive organs.

The family Orchidaceae, one of the largest families of flowering plants, has diverse life forms, life histories, habitats, morphology, and physiology (Zhang et al., 2018). Furthermore, orchids are well known for their ornamental flowers, which have long floral lifespans (Zhang et al., 2018). Orchids bear inflorescences with one or more flowers (Chen et al., 2009), and show great diversity in floral number and size (De, 2020). In orchids, the flower peduncle plays an important role in mechanical support, water transport, and nutrient transfer. Previous research in orchids has mainly focused on the physiology of vegetative organs (Zhang et al., 2018) and pollination biology (Waterman and Bidartondo, 2008). However, little is known about the allometric correlation among reproductive organ sizes in the family.

In the present research, we assessed the correlations between the number and the size of inflorescences, flowers, and leaves of 26 orchid species with various life forms. We asked three specific questions: (1) are there differences in the number and area from flower and leaf between orchid species with different life forms; (2) do orchid species with larger leaf area have larger inflorescence traits; and (3) do orchid species with large-diameter peduncles bear more and larger flowers concurrently than orchids with small peduncles? Our aims were to understand the development and allometry of reproductive and vegetative organs in Orchidaceae under natural selection.

## Materials and Methods

### Plant Materials

**Figure 1:**
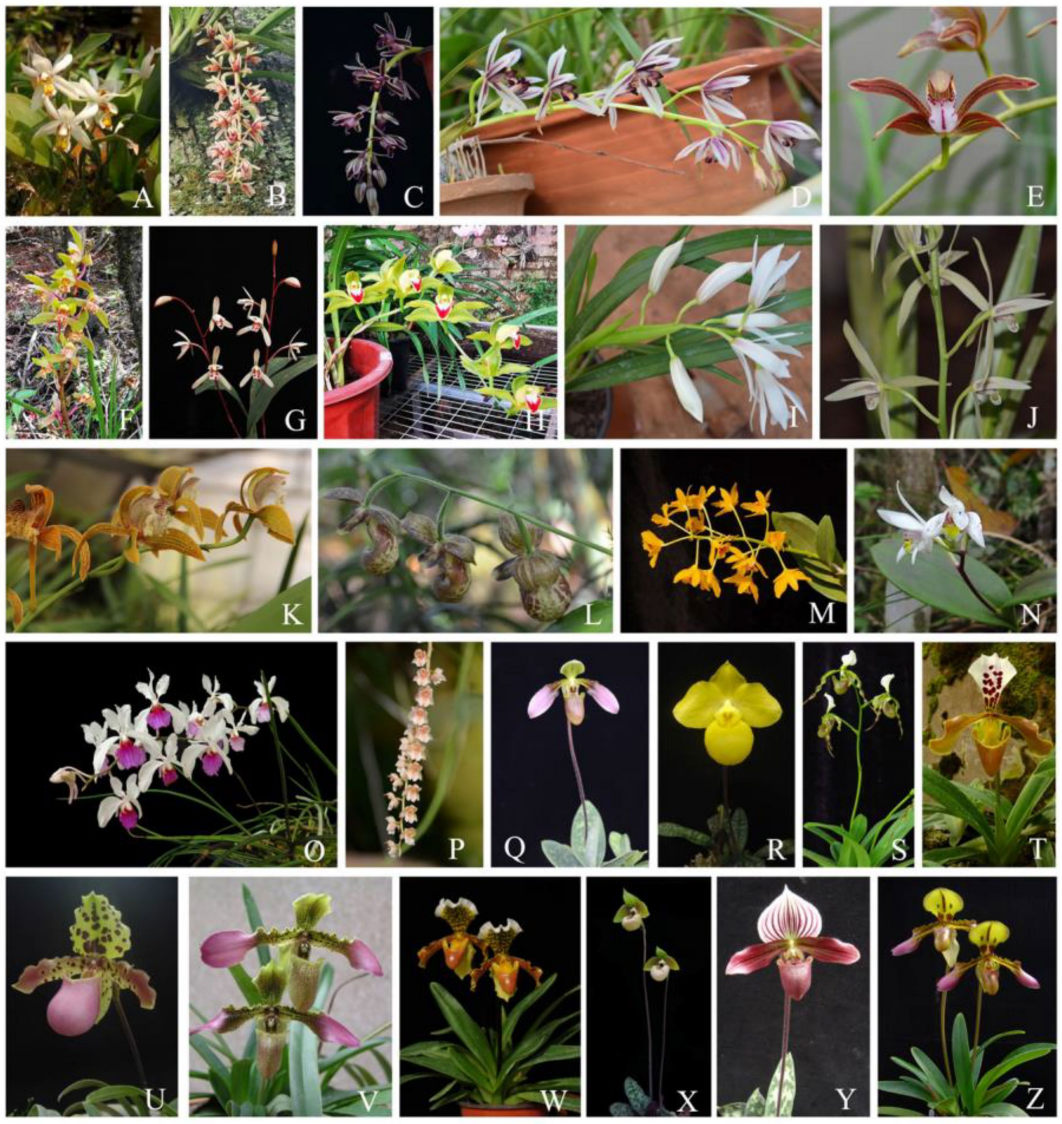
Orchid species studied in the present study. **(A)** *Coelogyne nitida*; **(B)** *Cymbidium aloifolium*; **(C)** *C. bicolor*; **(D)** *C. dayanum*; **(E)** *C. erythraeum*; **(F)** *C. faberi*; **(G)** *C. lancifolium*; **(H)** *C. lowianum*; **(I)** *C. mastersii*; **(J)** *C. sinense*; **(K)** *C. tracyanum*; **(L)** *Cypripedium subtropicum*; **(M)** *Dendrobium chrysotoxum*; **(N)** *Eria coronaria*; **(O)** *Holcoglossum kimballianum*; **(P)** *Pholidota chinensis*; **(Q)** *Paphiopedilum appletonianum*; **(R)** *P. armeniacum*; **(S)** *P. dianthum*; **(T)** *P. gratrixianum*; **(U)** *P. henryanum*; **(V)** *P. hirsutissimum*; **(W)** *P. insigne*; **(X)** *P. malipoense*; **(Y)** *P. purpuratum*; **(Z)** *P. tigrinum*. Erect raceme **(A,F,G,J,N,Q–Z)**; pendulous raceme **(B–D,P)**; arching raceme **(E,H,I,K–M,O)**.

We examined the traits of mature inflorescences, flowers, and leaves of 26 orchid species from 8 genera (*Coelogyne*, *Cymbidium*, *Cypripedium*, *Dendrobium*, *Eria*, *Holcoglossum*, *Pholidota*, and *Paphiopedilum*). Although all the studied species in this study are from the same family, Orchidaceae, their flower and leaf traits are different (Zhang et al., 2017). Here, 13 epiphytic orchids, 7 terrestrial orchids, and 6 facultative orchids of those studied orchid species were selected (Table 1). The inflorescences in these genera differ significantly (Figure 1) including erect, arching, or pendulous racemes from one flower up to many (up to 42) flowers. Inflorescences produced at the apical end of shoots are called terminal, the others arising from nodes near the base of pseudobulbs or leaf axils are lateral. The inflorescences of orchids are mostly terminal or lateral racemes (Arditti, 1992). Healthy, recently opened flowers and fully expanded leaves were collected from 3 to 6 individuals per species from the plants grown in a greenhouse at the Kunming Institute of Botany, Chinese Academy of Sciences (25°01' N; 102°41' E), under 30–40% full sunlight and temperatures of 20–25°C.

**Table 1:** Ecological and phenological traits of the studied orchid species.

Species	Life form	Number of flowers	Flowering period	Habitat	Altitude (m)
*Coelogyne nitida*	Epiphytic	2–3	March	On trees in the forest	1,400–2,700
*Cymbidium aloifolium*	Epiphytic	15–35	April–May	On trees or rocks in the forest	100–1,100
*Cymbidium bicolor*	Epiphytic	10–20	March–April	On trees in the forest	1,600
*Cymbidium dayanum*	Epiphytic	5–9	August–December	On trees in the forest	300–1,600
*Cymbidium erythraeum*	Epiphytic	3–7	October–January	On trees or rocks in the forest	1,400–2,800
*Cymbidium faberi*	Terrestrial	5–11	March–May	Understory	700–3,000
*Cymbidium lancifolium*	Facultative	2–6	May–August	Understory or rocks	300–2,200
*Cymbidium lowianum*	Epiphytic	10–20	April–May	On trees in the forest	1,300–1900
*Cymbidium mastersii*	Epiphytic	2–5	October–December	On trees or rocks in the forest	1,600–1800
*Cymbidium sinense*	Terrestrial	10–20	October–March	Understory	300–2000
*Cymbidium tracyanum*	Epiphytic	>10	September–December	On trees in the forest	1,200–1900
*Cypripedium subtropicum*	Terrestrial	-7	July	Understory	1,400
*Dendrobium chrysotoxum*	Epiphytic	>2	March–May	On trees or rocks in the forest	520–1,620
*Eria coronaria*	Epiphytic	2–6	May–June	On trees or rocks in the forest	1,300–2000
*Holcoglossum kimballianum*	Epiphytic	>2	November	On trees in the forest	1,000–1,630
*Pholidota chinensis*	Epiphytic	>20	April–May	On trees or rocks in the forest	1,500
*Paphiopedilum appletonianum*	Terrestrial	1	January–May	Understory	300–1,200
*Paphiopedilum armeniacum*	Facultative	1	March–May	Rocky or in crevices of rocks	1,400–2,250
*Paphiopedilum dianthum*	Epiphytic	2–4	September–November	On trees or rocks in the forest	550–2,250
*Paphiopedilum gratrixianum*	Terrestrial	1	September–December	Understory	1800–1900
*Paphiopedilum henryanum*	Facultative	1	September–November	On the grass slope of the edge of forest	900–1,300
*Paphiopedilum hirsutissimum*	Facultative	1	April–May	Understory	300–1,500
*Paphiopedilum insigne*	Terrestrial	1	October–December	On grassy and rocky slopes	1,200–1,600
*Paphiopedilum malipoense*	Terrestrial	1	January–April	Understory	800–1,000
*Paphiopedilum purpuratum*	Facultative	1	June–September	Understory or on rocks	1,200–1,500
*Paphiopedilum tigrinum*	Facultative	1	May–August	On trees or rocks in the forest	1,200–2,200

### Measurements of Inflorescence and Flower and Leaf Area

Whole flowering plants were selected, leaf number (LN) per flowering plant was recorded. The inflorescences and leaves of each collected species were excised in the morning, sealed in plastic bags, and immediately transported to our nearby laboratory. The inflorescence was selected to measure the lengths of inflorescence and to record the number of flowers (FN) when the top flower of the inflorescence was fully expanded. Inflorescence length (IL) was the length from the base of the inflorescence peduncle to the apex of the highest opened flower. Inflorescence length was measured using a ruler. The diameter of the peduncle (PD) was the average of two diameters which were measured along the major axis and the short axis with a vernier caliper. The newly opened flowers from the inflorescences were used to measure the floral area. Flowers (petals, sepals, and labellum) and leaves were cut into several sections, as they are uneven and tridimensional, to ensure they are flattened. The individual floral area was a total area corresponding to the sum of sepal, petal, and labellum. Individual floral area (IFA) and individual leaf area (ILA) were then determined with a Li-Cor 3000A area meter (Li-Cor, Inc., Lincoln, NE). Here, flower and leaf area were estimated: the total flower area (TFA) per inflorescence and total leaf area (TLA) per plant were estimated as the product of FN and IFA, and LN and ILA, respectively. Subsequently, the inflorescences, flowers, and leaves were oven-dried at 70°C for 48h to obtain their dry weights (DW). Flower dry mass per unit area (FMA, gm^−2^) was calculated as FDW/IFA, and leaf dry mass per unit area (LMA, gm^−2^) was calculated as LDW/ILA.

### Statistical Analysis

Differences between leaf and flower traits were analyzed using Tukey’s *post hoc* test after testing for normality and homogeneity of variances. All tests were made at a probability level of 5%. All statistical analyses were performed using SPSS 16 (SPSS, Chicago, IL).

## Results

**Figure 2:**
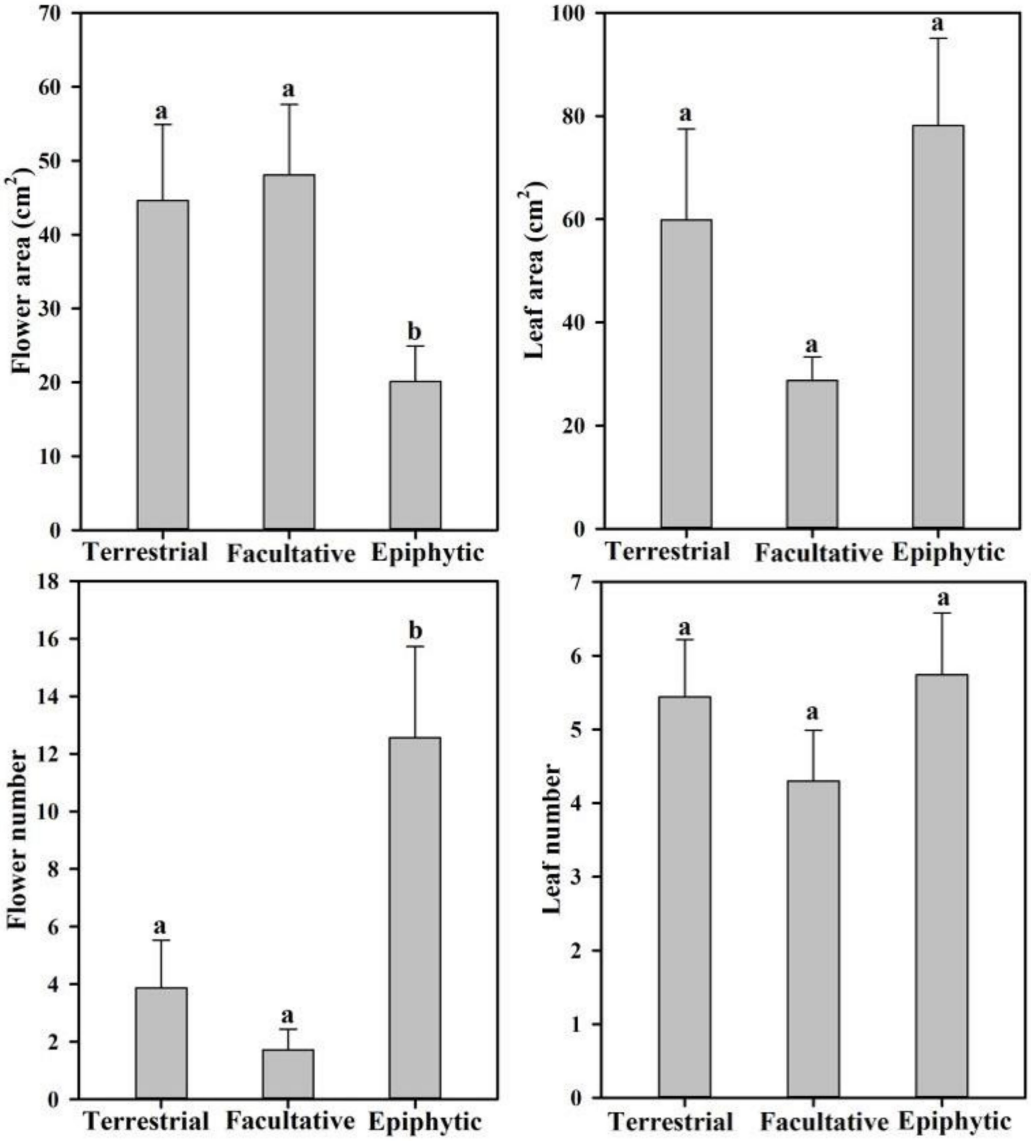
Differences in the number and area of flower and leaf from 26 terrestrial, facultative, and epiphytic orchid species.

In order to reveal whether there are differences in leaf and flower traits of orchids with different life forms, 10 traits associated with inflorescences, flowers, and leaves were examined across the 26 orchid species sampled (Tables 2 and 3). Statistical analysis showed that flower number and area differed significantly between orchid life forms (Figure 2). Epiphytic species had more flowers per inflorescence (12.56±3.17) than terrestrial species (3.87±1.66; *p*=0.04). Furthermore, the flowers of epiphytic species had smaller area (20.09±4.84cm^2^) than those of terrestrial species (44.63±10.26cm^2^; *p*=0.02). Facultative species had significantly fewer flowers (1.72±0.72) than did epiphytic species (*p*=0.02), but flower number did not significantly differ between facultative and terrestrial species (*p*=0.66). Flower area was significantly larger in facultative species (48.10±9.53cm^2^) than in epiphytic species (*p*=0.02), but it was not significantly different between facultative species and terrestrial species (*p*=0.78).

**Table 2:** Measured traits and units for inflorescences, flowers, and leaves from the studied orchid species.

Traits	Abbreviation	Unit	Mean±SE	Min	Max	CV (%)
Leaf number	LN	No.	5.53±0.52	2.00	11.33	48.43
Individual leaf area	ILA	cm^2^	64.67±10.17	14.63	226.30	80.17
Total leaf area	TLA	cm^2^	420.60±88.68	57.75	1764.89	107.52
Leaf dry mass per unit area	LMA	gm^−2^	130.44±9.68	42.47	294.87	37.86
Peduncle diameter	PD	mm^2^	3.63±0.39	1.39	10.70	55.48
Inflorescence length	IL	cm	39.57±4.53	10.18	90.20	58.40
Flower number	FN	No.	7.96±1.89	1.00	41.83	120.89
Individual floral area	IFA	cm^2^	34.81±4.93	0.90	84.81	70.20
Total floral area	TFA	cm^2^	146.45±32.54	32.50	678.15	113.29
Floral dry mass per unit area	FMA	gm^−2^	39.04±2.56	15.33	70.49	33.37

CV, coefficient of variation.

**Table 3:** The leaf and floral traits in the 26 orchid species studied.

Species	N	LN	ILA	TLA	LMA	PD	IL	FN	IFA	TFA	FMA
*Coelogyne nitida*	3	2.00±0.00	37.21±2.35	74.43±4.70	107.28±5.59	1.39±0.10	21.22±2.12	4.33±0.67	16.95±0.16	73.25±10.80	41.24±3.77
*Cymbidium aloifolium*	6	4.17±0.17	93.26±7.24	393.66±47.61	253.11±7.98	5.66±0.18	58.45±3.32	31.17±1.40	5.78±0.31	180.45±13.19	49.01±1.64
*Cymbidium bicolor*	6	6.33±0.33	57.67±3.09	366.40±31.09	294.87±15.42	3.63±0.07	21.28±1.10	12.83±0.91	5.07±0.09	65.11±4.82	49.40±1.46
*Cymbidium dayanum*	3	8.33±0.33	87.37±0.22	728.26±31.06	114.96±1.54	2.96±0.22	21.17±2.97	7.00±1.53	7.94±0.07	55.39±11.64	36.29±4.21
*Cymbidium erythraeum*	5	9.60±0.51	47.79±11.61	478.73±140.19	90.11±1.90	3.38±0.30	67.46±7.12	8.20±1.16	13.39±0.81	110.79±17.51	47.71±3.36
*Cymbidium faberi*	5	10.00±0.00	92.27±5.78	922.67±57.82	124.48±5.68	7.52±0.12	45.61±0.50	10.00±0.58	39.82±1.60	396.72±14.11	44.19±2.59
*Cymbidium lancifolium*	6	2.50±0.22	37.58±2.67	94.40±12.05	98.66±4.73	2.46±0.08	20.97±1.90	5.33±0.42	6.08±0.33	32.50±3.13	28.63±0.59
*Cymbidium lowianum*	4	6.50±1.04	175.23±29.33	1212.89±357.96	125.65±6.78	6.25±0.28	85.43±5.98	13.00±1.29	43.84±0.42	570.91±60.32	70.49±4.51
*Cymbidium mastersii*	3	11.33±1.20	75.58±4.18	866.62±134.83	117.43±4.62	4.25±0.27	27.23±3.69	5.00±1.15	24.42±0.65	122.18±27.78	31.30±1.32
*Cymbidium sinense*	5	6.00±0.32	118.50±8.98	705.89±49.03	126.18±5.76	4.06±0.35	52.76±4.52	12.60±0.93	10.26±0.83	130.73±16.78	33.71±1.57
*Cymbidium tracyanum*	3	8.00±1.00	226.30±25.36	1764.89±81.24	112.92±7.17	10.70±0.55	90.20±5.46	13.67±0.33	49.65±2.49	678.15±34.80	60.44±4.38
*Cypripedium subtropicum*	6	9.20±0.20	133.17±2.38	1223.60±14.62	42.47±1.84	4.24±0.42	28.48±4.35	6.60±1.36	47.55±2.92	322.83±74.10	23.46±1.34
*Dendrobium chrysotoxum*	6	3.17±0.17	24.14±3.65	75.98±10.96	117.83±4.37	3.12±0.12	16.71±1.23	11.33±0.67	16.74±0.88	190.90±16.43	41.17±1.00
*Eria coronaria*	5	2.20±0.20	52.89±2.52	115.22±7.84	142.09±2.52	2.33±0.09	10.18±0.56	4.40±0.40	9.09±0.25	39.96±3.59	30.27±0.37
*Holcoglossum kimballianum*	5	6.60±0.40	14.63±1.04	97.58±11.89	152.00±8.14	1.99±0.15	34.04±5.58	8.00±1.14	15.11±0.92	124.28±22.90	15.33±0.95
*Pholidota chinensis*	6	2.00±0.00	33.12±2.08	66.23±4.15	103.08±7.32	2.05±0.10	24.00±0.88	41.83±1.30	0.90±0.03	37.41±1.09	18.33±1.51
*Paphiopedilum appletonianum*	6	3.83±0.17	23.09±5.14	89.16±21.27	123.82±10.16	2.25±0.10	47.70±3.31	1.00±0.00	39.16±2.46	39.16±2.46	40.03±1.86
*Paphiopedilum armeniacum*	6	3.67±0.20	19.65±1.55	71.45±5.86	113.08±3.47	2.44±0.10	48.60±3.80	1.00±0.00	74.64±4.09	74.64±4.09	20.74±0.66
*Paphiopedilum dianthum*	5	4.40±0.51	91.17±5.75	397.09±42.66	178.80±8.64	4.28±0.14	38.62±2.49	2.60±0.24	52.24±1.57	135.98±13.80	53.94±1.90
*Paphiopedilum gratrixianum*	5	4.00±0.00	27.90±3.51	111.62±14.05	144.31±14.11	2.50±0.11	22.60±1.71	1.00±0.00	57.45±4.72	57.45±4.72	34.76±2.74
*Paphiopedilum henryanum*	5	3.40±0.24	17.35±3.27	57.75±9.19	133.12±6.04	2.30±0.11	19.76±1.56	1.00±0.00	48.81±1.75	48.81±1.75	29.45±1.61
*Paphiopedilum hirsutissimum*	6	6.8 3±0.54	43.42±8.30	283.88±47.62	139.72±3.45	3.37±0.25	35.10±1.80	1.00±0.00	52.97±3.10	52.97±3.10	54.43±2.34
*Paphiopedilum insigne*	5	3.60±0.24	49.01±4.03	178.74±23.82	117.83±8.92	3.22±0.08	26.60±2.36	1.00±0.00	76.18±4.96	76.18±4.96	42.37±1.70
*Paphiopedilum malipoense*	6	6.67±0.21	48.80±1.25	324.38±7.49	125.90±8.23	2.65±0.15	94.01±4.14	1.00±0.00	84.81±3.81	84.81±3.81	36.32±1.23
*Paphiopedilum purpuratum*	5	6.00±0.32	19.35±1.95	116.50±13.46	88.16±3.38	2.27±0.06	31.28±0.95	1.00±0.00	43.48±2.22	43.48±2.22	36.97±1.47
*Paphiopedilum tigrinum*	5	3.40±0.24	34.92±2.13	117.62±7.30	103.53±2.39	3.03±0.04	39.44±1.90	1.00±0.00	62.63±4.33	62.63±4.33	45.14±1.44

N, sample number; LN, leaf number; ILA, individual leaf area; TLA, total leaf area; LMA, leaf dry mass per unit area; PD, peduncle diameter; IL, inflorescence length; FN, flower number; IFA, individual floral area; TFA, total floral area; FMA, floral dry mass per unit area. Each value is mean±SE.

**Figure 3:**
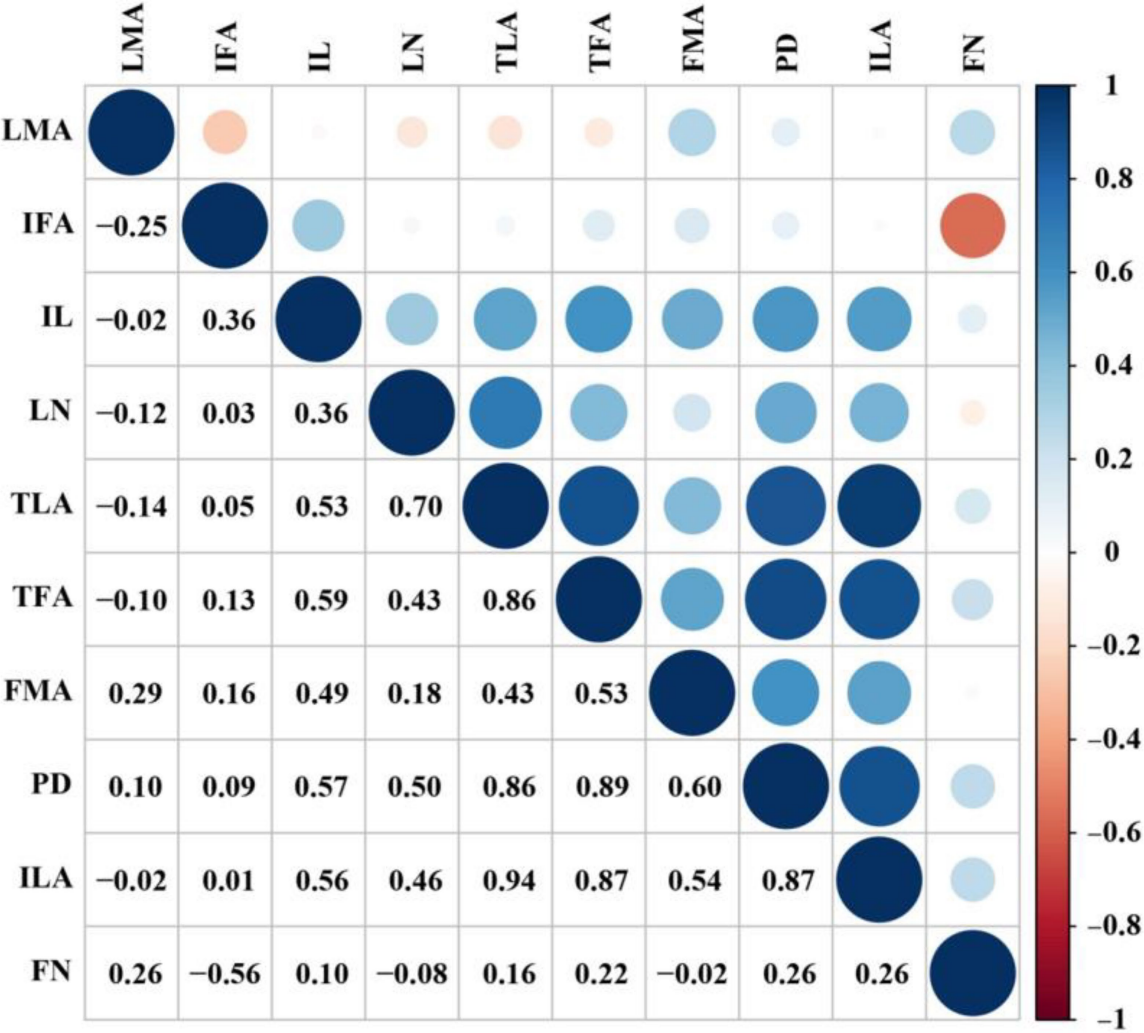
Correlations among inflorescence, floral, and leaf traits of the 26 orchids studied. Circle sizes represent the significance (upper right of the diagonal) and correlation coefficient (lower right of the diagonal). LN, leaf number; ILA, individual leaf area; TLA, total leaf area; LMA, leaf dry mass per unit area; PD, peduncle diameter; IL, inflorescence length; FN, flower number; IFA, individual floral area; TFA, total floral area; FMA, floral dry mass per unit area.

To further reveal the correlation between leaf traits and inflorescence traits, we found they were significantly correlated among them (Figure 3). For example, individual leaf area was positively correlated with peduncle diameter, inflorescence length, total floral area, and floral dry mass per unit area. Similarly, total leaf area was positively correlated with peduncle diameter, inflorescence length, total floral area, and floral dry mass per unit area.

The correlation among peduncle diameter, inflorescence length, flower number, individual floral area, total floral area, and floral dry mass per unit area was also analyzed in inflorescences (Figure 3). Peduncle diameter was positively correlated with inflorescence length, total floral area, and floral dry mass per unit area. However, flower number was negatively correlated with individual floral area. In addition, inflorescence length was positively correlated with total floral area and floral dry mass per unit area, while not correlated with individual floral area and flower number.

## Discussion

Different leaf and flower traits of various life forms are adapted to special habitats. Compared with terrestrial habitats, epiphytic habitats are stressed by water and nutrients (Benzing, 1990). Therefore, epiphytic species have higher velamen thickness, stomatal density, and leaf vein density than terrestrial species to add nutrients’ absorption and to reduce water loss (Zhang et al., 2012; Zotz and Winkler, 2013). However, the comparative study on flower traits between terrestrial and epiphytic orchid species is still lacking. In our study, we found that epiphytic species have lower flower area, while having a higher flower number than those of terrestrial species. We speculate that these differences may not only correlate to the water status of the flower but also correlate to the pollinator activities (Roddy and Dawson, 2012; Teixido and Valladares, 2014).

Leaf size and inflorescence size may be correlated in various plant species (Midgley and Bond, 1989). We also found that leaf area was positively correlated with peduncle diameter and inflorescence length. The larger leaf area can apply enough resources to construct the thicker peduncle diameter, larger inflorescence length, and flower area (Pleasants and Zimmerman, 1990). This correlation also indicated that there is a significant trade-off between leaf area and flower traits. To some extent, the allometry correlation between leaf area and flower traits contributed to the developmental or genetic constraints (Ackerly and Donoghue, 1998; Lambrecht and Dawson, 2007; Steven et al., 2019).

We found that inflorescence length was correlated with peduncle diameter and total floral area (Figure 3), but not with individual floral area or flower number. This correlation can also be found between peduncle diameter and total floral area or floral dry mass per unit area, which implies more biomass investment to the peduncle with the increase of total floral area as the correlation between petiole lamina size and size (Fan et al., 2017). Our findings coincide with the allometric correlations in other taxa. For example, a previous work on *Leucadendron* (Proteaceae) showed that inflorescence length is positively correlated with stem thickness (Midgley and Bond, 1989). Inflorescence architecture is closely related to the arrangement of each flower on an inflorescence (Prusinkiewicz et al., 2007). However, thus far, the study on the correlation between peduncle diameter and inflorescence architecture is still lacking. In our study, we found no significant correlation between peduncle diameter and inflorescence architecture. For example, compared with *C. lancifolium*, which has a thinner peduncle but erect racemes, *C. aloifolium* has a thicker peduncle but pendulous racemes. These results indicate that the inflorescence architecture may be related to the specific habitat (Schoen and Dubuc, 1990). Taken together, these findings imply that inflorescences provide water, nutrients, and mechanical support to flowers, which is analogous to what twigs (stems) provide to leaves (Niklas and Enquist, 2003; Fan et al., 2017). Likewise, the allometric relationship between leaf area (mass) and petiole area (mass) was found, indicating that larger leaves invest a higher fraction of biomass in the petiole than smaller leaves (Fan et al., 2017). Our finding was also important in horticultural applications. Previous studies have shown that crosses between orchids with different numbers of flowers, but similar peduncle diameters, can produce hybrids with intermediate flower numbers such as *Orchis pauciflora* and *O. mascula* (Cozzolino et al., 2006) or *Anacamptis*× *albuferensis* (Bateman and Hollingsworth, 2004). In contrast, crosses between orchids with similar flower numbers but different peduncle diameters produce offspring with thicker peduncle diameters and more flowers than their parentals (Yan et al., 2017).

The absence of significant correlations between flower number or flower area with leaf number or leaf area strongly suggests that orchid flower and leaf traits are two functional traits independent of evolution (Zhang et al., 2017), which might result from not only biotic and abiotic pressures but also the functions of different organs. This differentiation is consistent with the prevalence of differing selective pressures upon fundamental function and genetic background of reproductive vs. vegetative organs (Juenger et al., 2005; Pélabon et al., 2011; Roddy et al., 2013). For example, leaf number reduces significantly under water stress (Descamps et al., 2020). Leaf area tends to be small in poor habitats (Yang et al., 2010). Different from leaves, in order to ensure successful reproduction, plants can regulate the flower number in specific environments (Prusinkiewicz et al., 2007). The flower number and area are significantly decreased with the increase of temperatures (Descamps et al., 2020). The positive correlation observed here between total floral area and total leaf area indicated the importance of the coordinating role of the size between reproductive and vegetative organs. Larger leaf area may assimilate more carbon, thus more carbon can be used in flowers (Lambrecht and Dawson, 2007). A previous study has suggested that larger flowers produce a better return for plant reproductive success and fitness than smaller flowers (Sargent et al., 2007). Larger flowers can receive more pollinators, and it seems probable that larger flowers enhance reproductive fitness in the plant-pollinator system (Galen, 1989; Teixido and Valladares, 2014). Studies on *Paphiopedilum* and *Cymbidium* species indicate that plants with larger and more flowers have more fruit sets (Bänziger, 1996; Cheng et al., 2007; Shi et al., 2007; Yu et al., 2008). For example, *P. dianthum*, which has twice the floral area of *P. villosum* and nearly three times the number of flowers, sets roughly eight times the amount of fruit (Bänziger, 1996). However, larger flowers may also increase construction and maintenance costs. Hence, future work should focus on addressing how plants trade-off between the size of flowers and physiological maintenance costs.

## Conclusion

Our study demonstrated that inflorescence length in orchids is correlated with peduncle diameter, total floral area, and individual and total leaf area. However, inflorescence length is not correlated with individual floral area or flower number. These results provide novel insights into the development and allometry of reproductive and vegetative organs in Orchidaceae under natural selection. Moreover, our findings are of broader significance to breeding new hybrid orchids.

## Data Availability Statement

The original contributions presented in the study are included in the article/supplementary material, further inquiries can be directed to the corresponding authors.

## Author Contributions

F-PZ, S-BZ, J-QF, and HH designed the study and conceived the manuscript. J-QF collected the samples and data. F-PZ conducted statistical analyses and wrote the first draft of the manuscript. All authors contributed to the article and approved the submitted version.

## Funding

This study was supported by the National Natural Science Foundation of China (31970361 and 31960224), the “Young Top Talents” of the Ten Thousand Talents Plan in Yunnan Province (YNWR-QNBJ-2018-337), Science Research of Yunnan Provincial Department of Education (2019J1068), Yunnan Applied Basic Research Project (2018FA016), the Project for Construction of International Flower Technology Innovation Center and Achievement Industrialization (2019ZG006), Yunnan Provincial Science and Technology Department-Applied Basic Research Joint Special Funds of Yunnan University of Chinese Medicine (202001AZ070001-041), the Project for Innovation Team of Yunnan Province (202105AE160012), and the Digital Development and Application of Biological Resources in Yunnan Province (202002AA100007).

## Conflict of Interest

The authors declare that the research was conducted in the absence of any commercial or financial relationships that could be construed as a potential conflict of interest.

## Publisher’s Note

All claims expressed in this article are solely those of the authors and do not necessarily represent those of their affiliated organizations, or those of the publisher, the editors and the reviewers. Any product that may be evaluated in this article, or claim that may be made by its manufacturer, is not guaranteed or endorsed by the publisher.
